# Research hotspots and trend of a emerging novel endoscopic technique of peroral endoscopic myotomy from 2010 to 2022: A bibliometric analysis

**DOI:** 10.1097/MD.0000000000035009

**Published:** 2023-09-08

**Authors:** Qingliang Zhu, Han Zhang, Shu Huang, Peiling Gan, Ruiyu Wang, Yan Peng, Muhan Lü, Xiaowei Tang

**Affiliations:** a Department of Gastroenterology, The Affiliated Hospital of Southwest Medical University, Luzhou, China; b Department of Gastroenterology, Zigong First People’s Hospital, Zigong, China; c Department of Gastroenterology, Lianshui County People’ Hospital, Huaian, China.

**Keywords:** achalasia, bibliometric analysis, CiteSpace, peroral endoscopic myotomy

## Abstract

Peroral endoscopic myotomy (POEM), which has been used to treat achalasia and other esophageal motility disorders for the past 10 years, has proven to be secure and efficient. Every year, more and more essays on this subject are published. We sought to investigate the global scientific outputs and hotspots of POEM produced by various nations, organizations, and authors. From 2010 to October 2022, there were 875 papers on POEM that were found in the Web of Science Core database. The bibliometric visualization analyses of nations/regions, institutions, authors, journals, references, and keywords were conducted by CiteSpace V.5.8.R3. Eight hundred seventy-five publications were included in this analysis. With 68 publications, Inoue H had the highest output. While Showa University in Japan was the most productive institution, the United States was the most productive nation. Among the journals, *Surgical Endoscopy* published the highest number of articles, followed by *Gastrointestinal Endoscopy* and *Endoscopy.* The top 10 keywords that appeared most frequently were achalasia, peroral endoscopic myotomy, POEM, myotomy, esophageal achalasia, dysphagia, heller myotomy, endoscopy, gastroparesis and peroral endoscopic myotomy. Seven frontiers, including meta-analysis, high-resolution esophageal manometry, geriatric patient, third space endoscopy, adverse event, endoscopic submucosal dissection, and gastric peroral endoscopic myotomy, had an impact on future research on POEM. The previous 10 years have seen a considerable rise in POEM research, and this trend will continue. The most recent research frontiers, which require more attention, are meta-analysis, high-resolution esophageal manometry, geriatric patient, third space endoscopy, adverse event, and gastric peroral endoscopic myotomy.

## 1. Introduction

Peroral endoscopic myotomy (POEM) was first reported in 2010 by Inoue et al^[1]^ in 17 achalasia patients. Since then, more and more studies on POEM have shown that it was a safe and effective minimally invasive treatment modality for achalasia.^[2–4]^ POEM also showed a favorable clinical response in other esophageal motility disorders and in a salvage treatment for achalasia after previously failed intervention.^[5,6]^ Besides, POEM had been continuously refined and modified, and led to advantageous derivatives such as peroral endoscopic tumor resection or submucosal tunneling endoscopic resection for resection of subepithelial tumors, Zenker POEM for Zenker diverticula, diverticular POEM for epiphrenic esophageal diverticula, and gastric POEM for refractory gastroparesis.^[7–10]^ Over the last decade, POEM had been expanded and developed. Despite an increasing research output in the POEM field, little is known about the scientific production related to POEM and a bibliometric analysis has not been published to date.

According to Dr Chaomei Chen, bibliometric research is a statistical and quantitative analytical technique that identifies the characteristics of publications and academic impact of journals, researchers, institutions, and countries within a research field.^[11]^ It can be used by researchers to spot trends and pinpoint new areas of study.^[11]^ Related studies such as endoscopic submucosal dissection,^[12]^ endoscopic retrograde cholangiopancreatography,^[13]^ and endoscopic ultrasound^[14]^ were conducted using this method. In order to provide references for future research direction and collaboration, we set out to clearly investigate the beginning and significant turning points of the research on POEM.

## 2. Methods

Because our data came from a public database, ethical approval was not required for our research.

### 2.1. Data source and search strategy

The Web of Science Core database, which is regarded as the most ideal database for bibliometric analysis, was used to conduct a thorough literature search. TS = (peroral endoscopic myotomy) OR TS = (peroral endoscopic myotomy) were the specific search algorithms that were applied. The search time for the literature was from 2010 to October 2022, and only original articles and reviews were allowed. In order to eliminate bias brought by frequent database renewal, we conducted a literature search and retrieved the raw data on October 23, 2022. The original procedure of retrieval eliminated all non-English literature. Finally, we eliminated redundant and unrelated articles from our study.

### 2.2. Analysis tool

In order to provide clinicians and researchers in this field with scientific and user-friendly support, CiteSpace V.5.8.R3 was chosen to perform the bibliometric analysis on the publications related to POEM by integrating information about countries/regions, authors, institutes, journals, citation, and keywords. CiteSpace is a Java application that combines information visualization techniques, bibliometrics, and data mining algorithms in an interactive visualization tool. It was developed by Dr Chaomei Chen (School of Information Science and Technology, Drexel University, Philadelphia, PA) and his team in 2004.^[15]^

### 2.3. Data analysis

As a test platform for CiteSpace, the datasets for the analysis of articles on POEM were created. Two thousand through 2022 was the time frame. Analysis of the cooperation networks (including authors, institutions, countries/regions, and journals), reference co-citations, cluster analysis of keywords that co-occur, and keywords burst detection were done. Depending on the type of study, the results were represented by various types of clusters in a node-circle network. The analysis objects are represented by the nodes. Each node’s circle’s color and thickness correspond to different time periods frequency. An indicator of a node’s prominence in a network, centrality is measured by its brilliant purple outer rings. High centrality is sometimes viewed as a turning point in a field.^[16]^ Future study directions were determined using keywords burst detection.^[17]^

## 3. Results

### 3.1. General data

**Figure 1.**
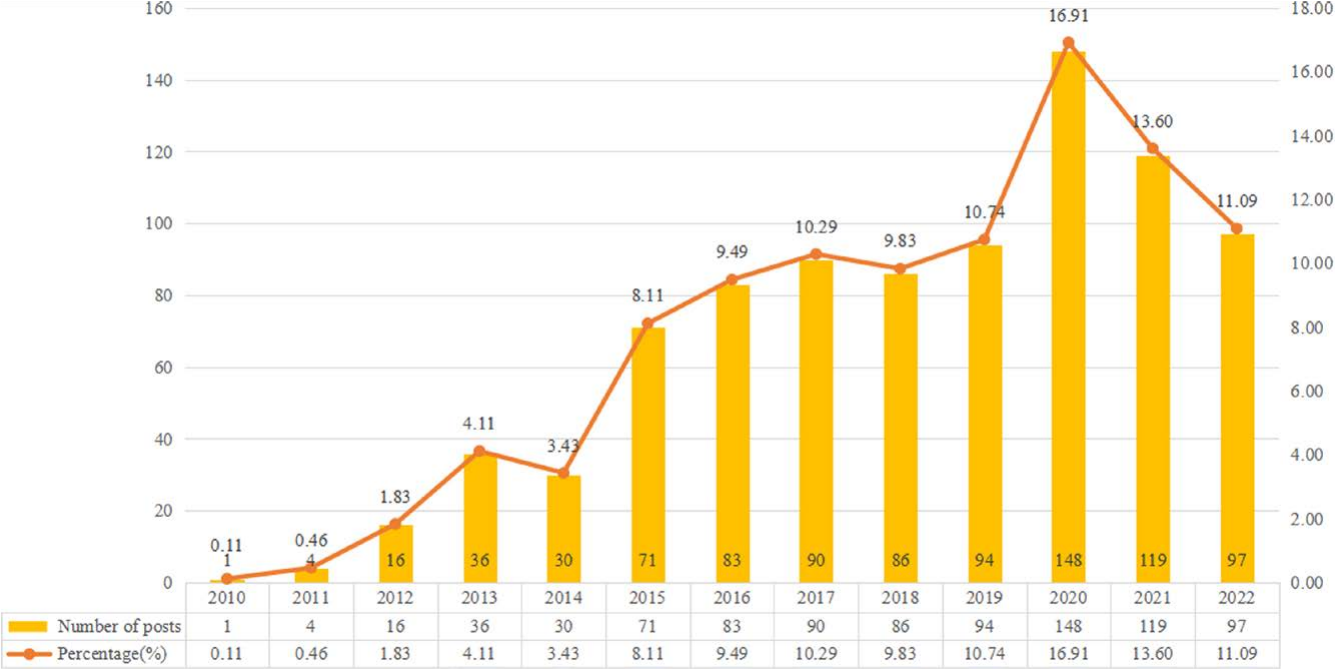
The number of POEM publications indexed by WoS CC, 2010–2022. POEM = peroral endoscopic myotomy, WOS = Web of Science.

The search strategy for POEM generated 1824 studies, including 670 original articles and 205 reviews, published in English between 2010 and 2022, after filtering out the duplicate records. Inoue et al firstly developed and performed POEM to treat achalasia patients in 2008, and then the first article in the field of POEM was published in 2010. The number of publications gradually and generally increased in the subsequent years. According to the publication years, the quantity of published articles on POEM increased significantly from 1 in 2010 to 148 in 2020 (Fig. 1).

### 3.2. Countries/regions

**Figure 2.**
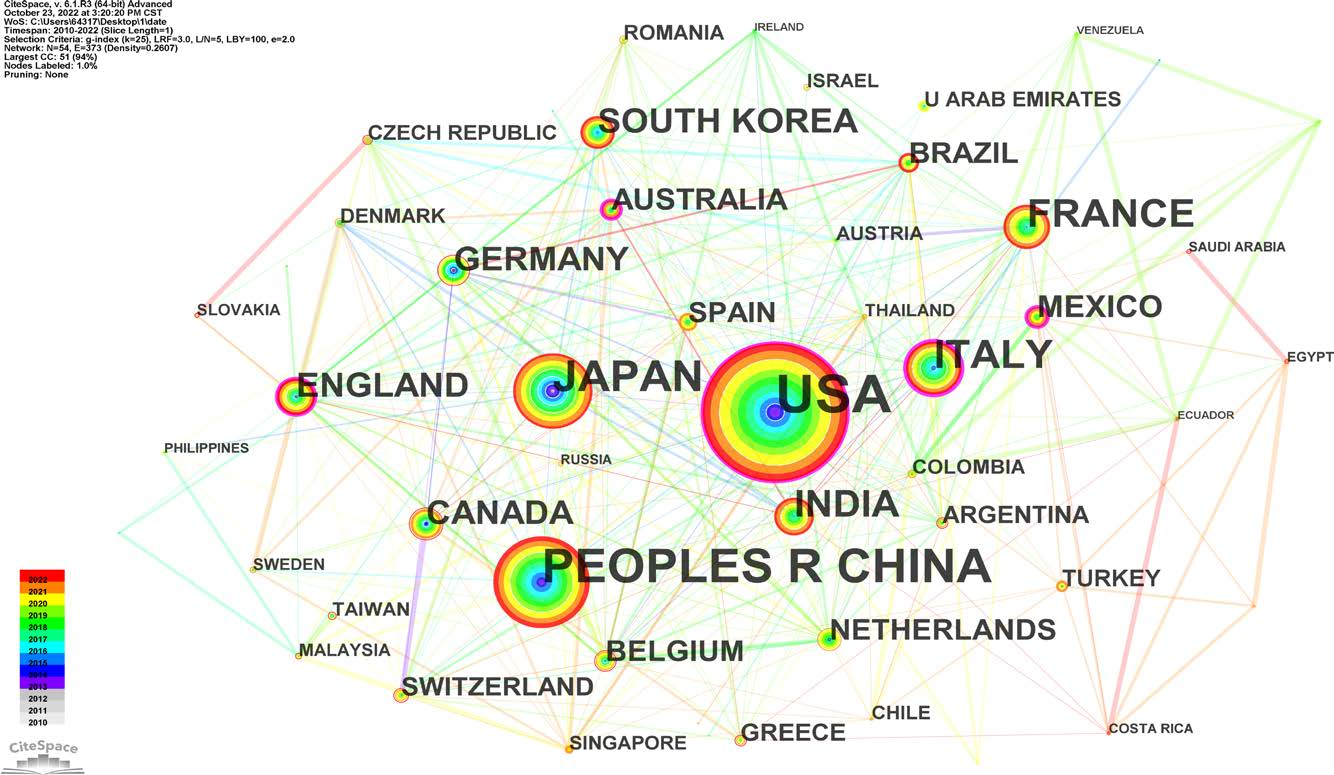
Map of countries/regions cooperative relations in research of POEM, 2010–2022. POEM = peroral endoscopic myotomy.

Figure 2 depicts the network of the producing nations and areas. The size of the circles indicates the number of publications made by the various nations and areas, and the closer the distance between 2 circles, the more cooperation there is between the various nations and locations. Higher centrality, which is often viewed as the field’s pivotal point, is denoted by a circle with a broader purple ring. In terms of publishing volume, the United States (387) came out on top among all relevant nations/regions, followed by the People’s Republic of China (188) and Japan (118). In the POEM research, the top 10 productive nations/regions were displayed in Table 1.

**Table 1:** The top 10 countries, institutions, and authors contributed to publications of POEM research.

Country	Articles (n)	Institution	Articles (n)	Author	Articles (n)
USA	387	Showa Univ	64	Inoue H	68
Peoples R China	188	Northwestern Univ	41	Khashab M	53
Japan	118	Fudan Univ	32	Pandolfino J	31
Italy	80	Johns Hopkins Med Inst	27	Zhou P	28
France	70	Univ Chicago	25	Zhang Y	26
India	55	Oregon Clin	22	Ujiki M	24
England	39	Asian Inst Gastroenterol	22	Li Q	24
South Korea	37	Niigata Univ	21	Sato H	24
Germany	34	Emory Univ	20	Onimaru M	23
Canada	34	Johns Hopkins Univ Hosp	18	Kumbhari V	22

POEM = peroral endoscopic myotomy.

### 3.3. Institutes

**Figure 3.**
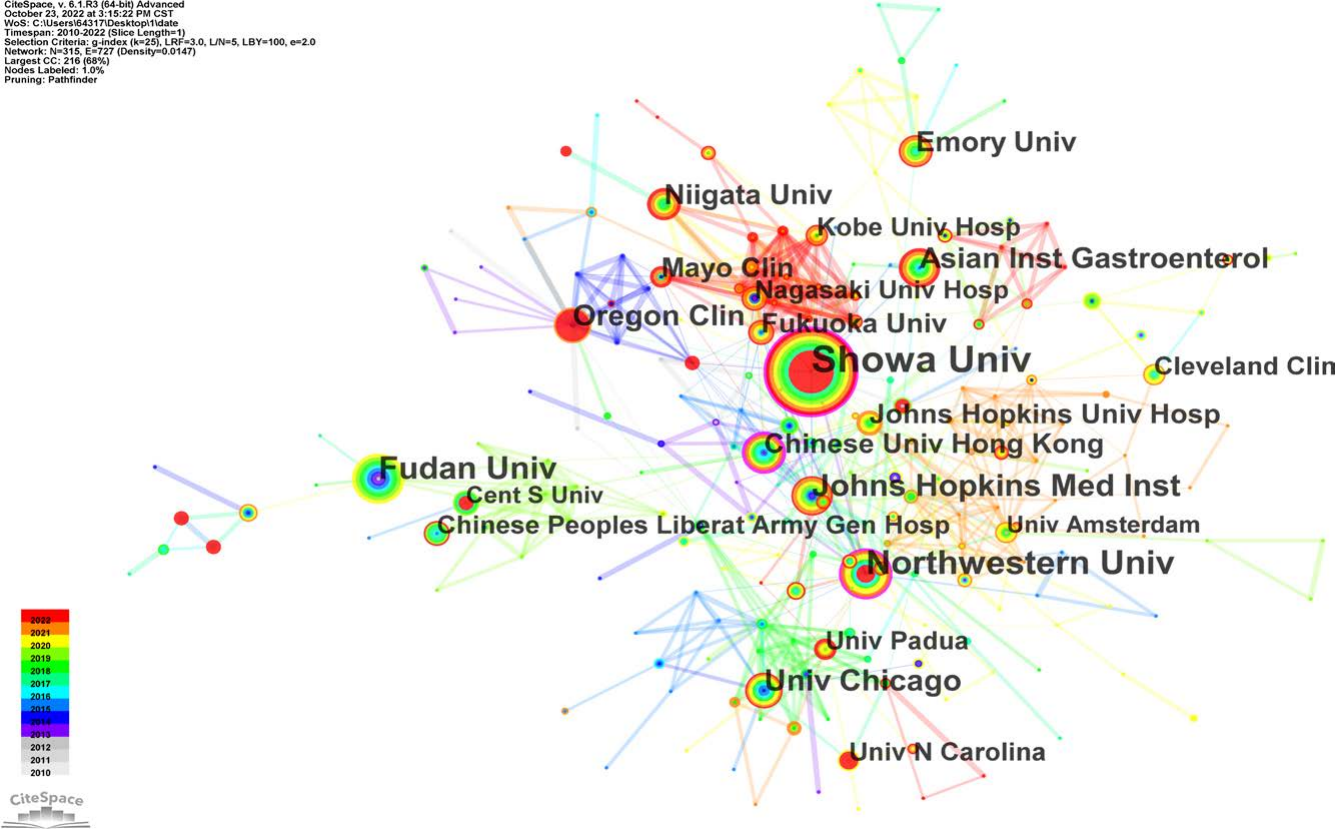
Map of institutes cooperative relations in research of POEM, 2010–2022. POEM = peroral endoscopic myotomy.

The main prolific co-institutes in the field of POEM were depicted in Figure 3. With a total of 64 published articles, Showa University was the most productive and well-known institution in this discipline, followed by Northwestern University (41 publications) and Fudan University (32 publications). It should be noted that the USA had 6 out of the top 10 most productive institutes, indicating that the nation had a strong position in the most recent POEM research (Table 1).

### 3.4. Authors

**Figure 4.**
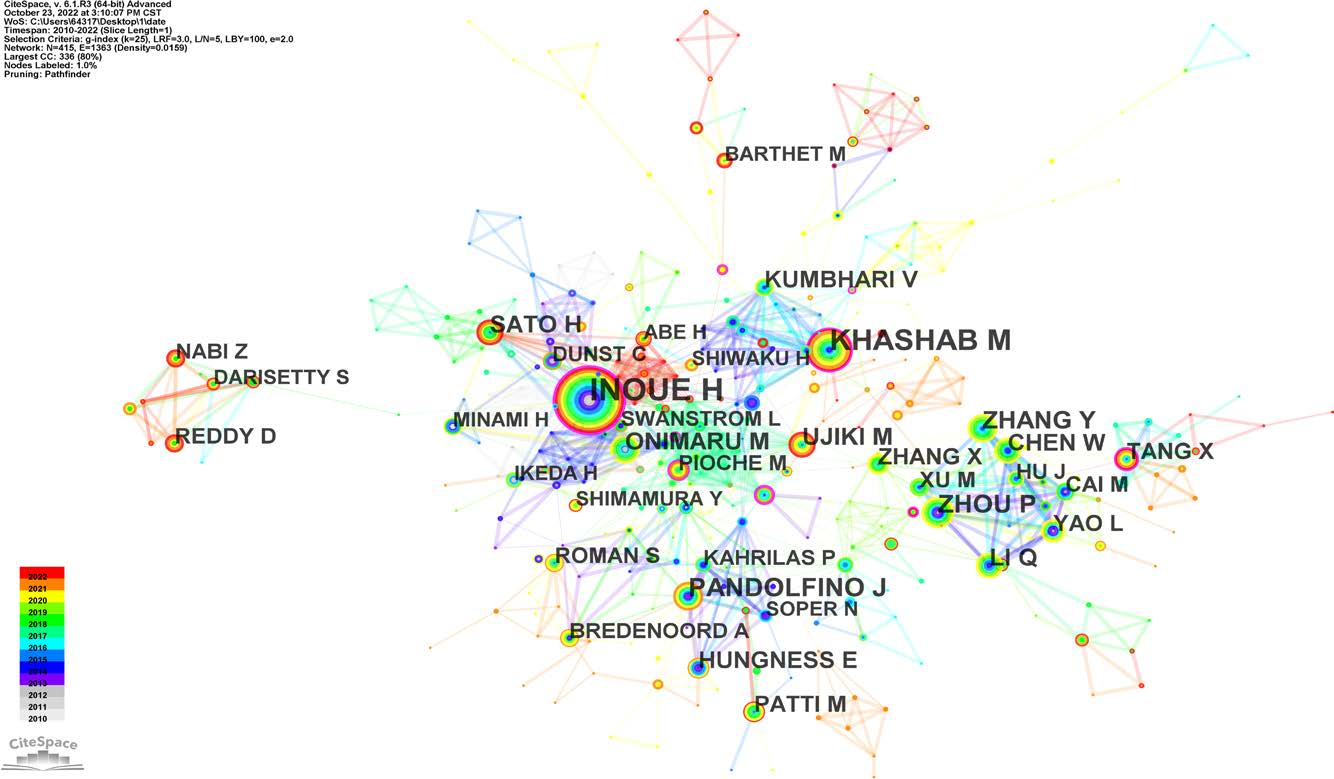
Co-authorship network map in research of POEM, 2010–2022. POEM = peroral endoscopic myotomy.

For the identification of potential cooperation between authors, the co-authorship was illustrated by a network map generated by CiteSpace (Fig. 4). Cooperation relationships are represented by connections among nodes. The thicker the connection is, the closer the cooperation is. Regarding the authors who were active, Inoue H ranked the first (68 publications), followed by Khashab (53 publications) MA and Pandolfino J (31 publications) (Table 1).

### 3.5. Reference co-citation and journals

**Figure 5.**
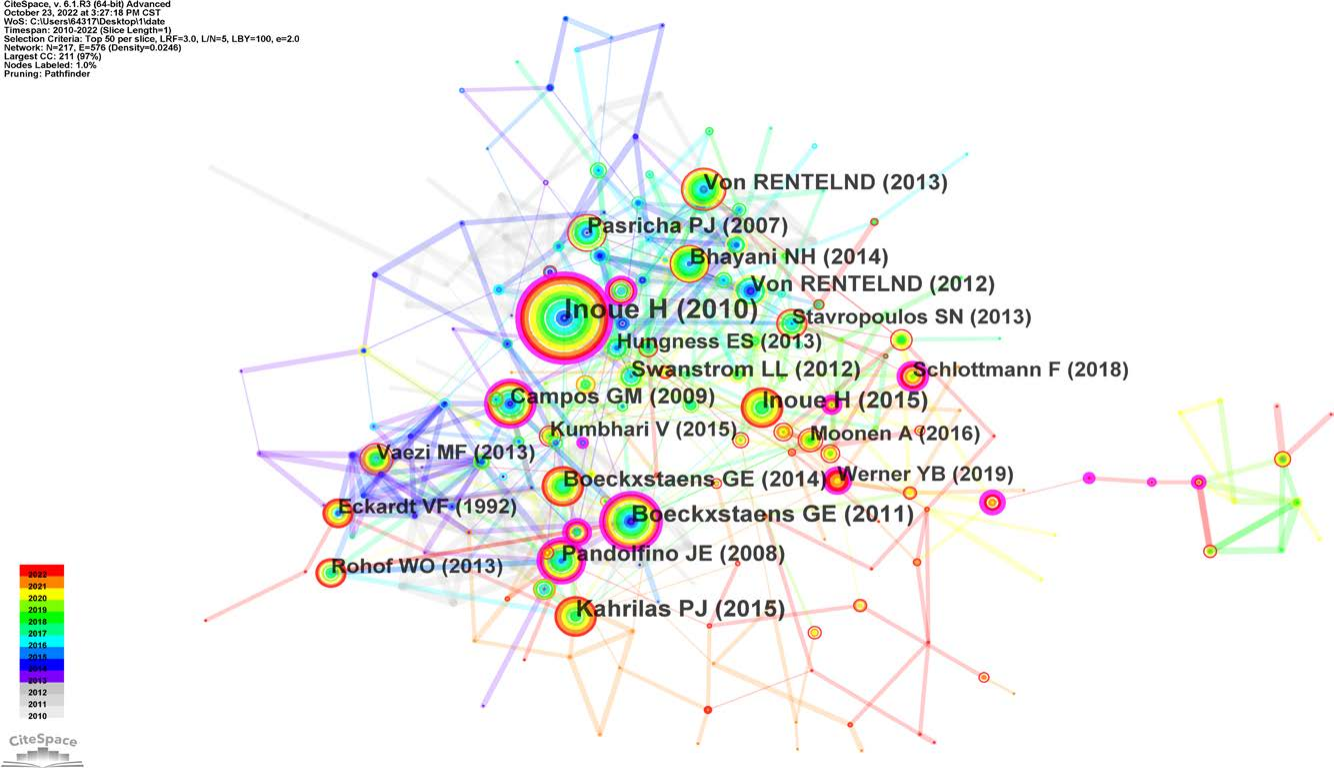
Map of reference co-citation related to research of POEM, 2010–2022. POEM = peroral endoscopic myotomy.

Visualization of the largest reference co-citation network was shown in Figure 5. The top ranked article by co-citation counts was Inoue H (2010), with citation counts of 530. The second 1 was Boeckxstaens GE (2011), with citation counts of 225, followed by Kahrilas PJ (2015) (Table 2). Table 3 lists the top 10 highly cited journals. The impact factors (IF) of the top 10 journals ranged from 1.766 to 10.396 (average of 5.188), with an IF > 5.000 in 4 journals. The highest 1 was *Surgical Endoscopy*, with 130 articles (IF, 2021 = 3.453), followed by the *Gastrointestinal Endoscopy* (62 articles, IF, 2021 = 10.396) and *Endoscopy* (45 articles, IF, 2021 = 9.776).

**Table 2:** Top 5 co-citation references related to POEM, 2010–2022.

Ranking	Frequency	Source	Cited reference	Representative author	Publication yr
1	530	Endoscopy	Peroral endoscopic myotomy (POEM) for esophageal achalasia	Inoue H	Inoue H
2	225	New Engl J Med	Pneumatic dilation versus laparoscopic heller myotomy for idiopathic achalasia	Li Q	Boeckxstaens GE
3	214	Neurogastroent Motil	The Chicago classification of esophageal motility disorders, v3.0	Sato H	Kahrilas PJ
4	200	J Am Coll Surgeons	PerOral endoscopic myotomy: a series of 500 patients	Onimaru M	Inoue H
5	167	Gastroenterology	Peroral endoscopic myotomy for the treatment of achalasia: an international prospective multicenter study	Kumbhari V	Von rentelnd

POEM = peroral endoscopic myotomy.

**Table 3:** The top 10 journals contributed to publications of POEM research.

Journal	Articles (n)	Impact factor (2021)
Surgical Endoscopy and Other Interventional Techniques	130	3.453
Gastrointestinal Endoscopy	62	10.396
Endoscopy	45	9.776
World Journal of Gastroenterology	29	5.374
Diseases of the Esophagus	28	2.822
Digestive Endoscopy	28	6.337
Journal of Gastrointestinal Surgery	27	3.267
Journal of Laparoendoscopic & Advanced Surgical Techniques	27	1.766
Neurogastroenterology and Motility	26	3.960
Journal of Neurogastroenterology and Motility	19	4.725

POEM = peroral endoscopic myotomy.

### 3.6. Keywords cluster analysis and burst detection

**Figure 6.**
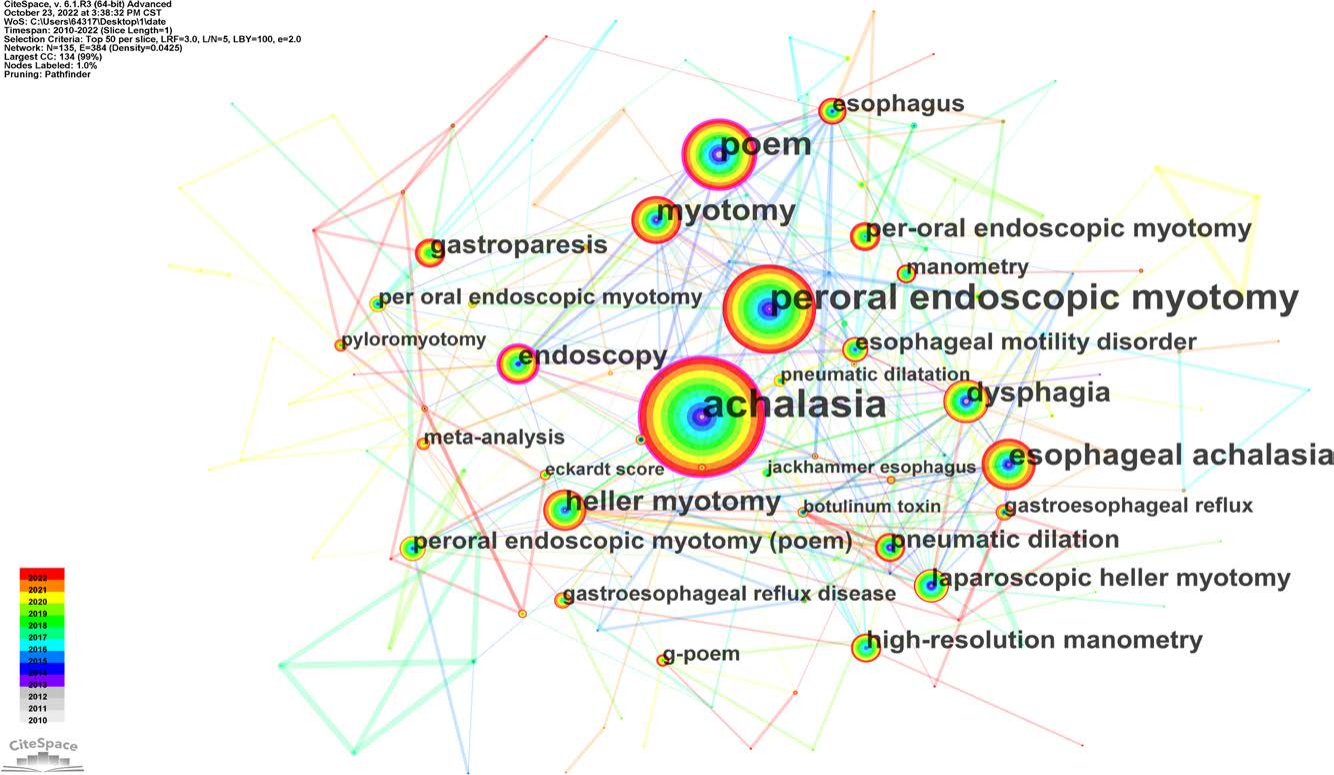
Map of keyword co-occurrence related to research of POEM, 2010–2022. POEM = peroral endoscopic myotomy.

**Figure 7.**
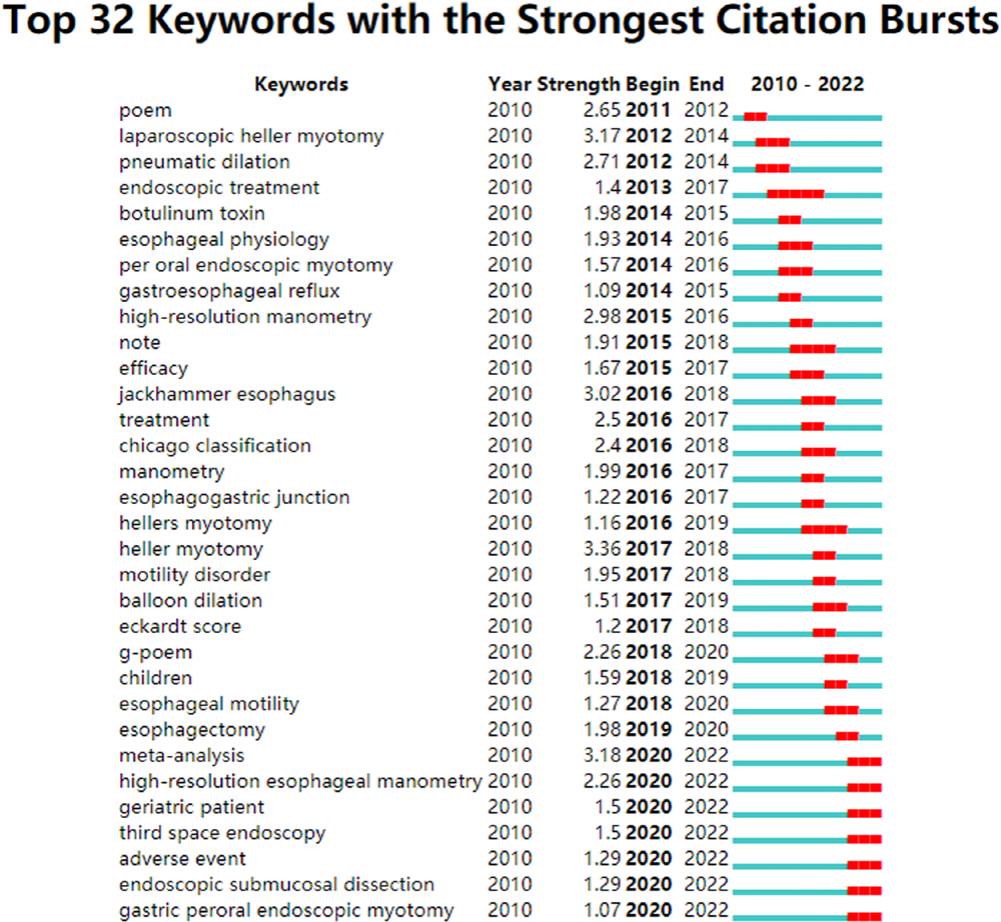
Keywords with the strongest citation bursts in published articles on POEM, 2010–2022. POEM = peroral endoscopic myotomy.

The hot regions in the literature can be found by analyzing the keywords. The top 10 terms that appeared most frequently were achalasia, peroral endoscopic myotomy, poem, myotomy, esophageal achalasia, dysphagia, heller myotomy, endoscopy, gastroparesis and peroral endoscopic myotomy. Figure 6 displays the results of a keyword co-occurrence study. The keywords are generalizations of the literature’s subjects. In a certain study field, keywords burst detection can find rapidly expanding themes that persist throughout time as well as those that only exist for a single year.^[18]^ Figure 7 displays the top 32 terms with the most significant bursts of citations in works on POEM. The strongest burst term from 2017 to 2018 was “heller myotomy,” with a burst strength of 3.36; it was followed by “meta-analysis” from 2020 to 2022 (3.18); “laparoscopic heller myotomy” (3.17) from 2012 to 2014; and “jackhammer esophagus” (3.02) from 2016 to 2018. Seven horizons, including meta-analysis, high-resolution esophageal manometry, geriatric patient, third space endoscopy, adverse event, endoscopic submucosal dissection, and gastric peroral endoscopic myotomy, have an influence on future research on POEM.

## 4. Discussion

In this study, we conducted a bibliometric analysis of the literature in the field of POEM published from 2010 to October 2022, and preliminarily explored the research status and predicted future research hot spots in this field. It was found that the first article in the field of POEM was published in 2010, and subsequently, researches in this field mushroomed, with the number of publications reaching a peak in 2020. After 10 years of development, POEM has gradually become the first-line therapy in the treatment of achalasia, and has derived a series of related endoscopic treatment techniques, which has brought revolutionary progress in the field of endoscopic treatment. Therefore, POEM is currently recognized as a better principle natural orifice transluminal endoscopic surgery procedure than surgical operation.

This study showed that Japan, as the first country to perform POEM, has many advantages in the field of POEM. For example, as the pioneer of POEM, Inoue H is the most published and influential author in this field. Inoue H first published study on POEM in 2010, “POEM for esophageal achalasia”,^[1]^ is the most cited reference in this field. Showa University in Japan is the most productive and influential institution. All these indicate that Japan has an important impact on the development of POEM. However, through the analysis of the number of publications by country/region, we found that Japan was not the country with the largest number of publications, but followed the United States and China. The United States is the country with the largest number of publications, which is inseparable from its developed economy and strong scientific research foundation. China has surpassed Japan to become the second country in the number of publications, which is closely related to Chinese large population base. China has become the country with the largest number of POEM, and Chinese endoscopists have become increasingly proficient in POEM technology. As a result, the United States and China are playing an increasingly important role in the field of POEM.

Keywords can simply and comprehensively summarize the main research content of the literature. Through keyword analysis, we can understand the research hot spots in a specific field and predict the future research trend. Through keyword co-occurrence analysis and keyword burst detection in this study, we found that achalasia, as an initial indication, is the most relevant keyword to POEM. However, on further analysis, we found keywords such as jackhammer esophagus, Z-POEM, G-POEM, esophageal diverticulum, and gastroparesis. With the continuous development of POEM, some derivative techniques have emerged, and these derivative techniques have been applied in clinical practice. For example, Z-POEM has been used in the treatment of Zenker diverticulum, with reported overall clinical success rates of 93%.^[19]^ G-POEM has also been reported for the treatment of refractory gastroparesis, and the success rate is impressive.^[20]^ However, more high-quality multicenter prospective studies with long-term follow-up are needed to further confirm their safety and efficacy. Combined with the bibliometric analysis of this study, D-POEM for esophageal diverticulum, Z-POEM for Zenker diverticulum, and G-POEM for refractory gastroparesis will be the research hot spots in the field of POEM in the future.

Besides, it is worth noting that meta-analysis is one of the most popular research spots from 2020 to 2022 with the burst strength of 3.18. Currently, dozens of meta-analyses on POEM have been published. Meta-analysis initially focused on the short-term safety and success of POEM.^[21,22]^ Then, the focus shifted to compare the efficacy between other traditional treatment methods for achalasia, such as laparoscopic Heller myotomy and pneumatic dilatation, and POEM.^[23,24]^ In addition, a comparison of the efficacy of various operation methods of POEM has gradually emerged, such as anterior versus posterior direction anterior versus posterior myotomy^[25]^ and short versus standard esophageal myotomy.^[26]^ At the same time, studies on the efficacy of POEM in the treatment of special populations such as the pediatric,^[27]^ the geriatric,^[28]^ the failed Heller myotomy patients,^[29]^ and other esophageal motility disorders patients^[30]^ are also gradually emerging. Recently, the efficacy of the derivative technology of POEM, such as D-POEM for esophageal diverticulum,^[31]^ Z-POEM for Zenker diverticulum,^[19]^ and G-POEM for refractory gastroparesis^[20]^ has gradually become a hot topic. In the future, with the continuous extension of follow-up time, meta-analysis of the long-term efficacy and safety of these operations will become the research direction.

In addition, we also found some important issues about POEM. POEM has become the first-line protocol recommended by current guideline for the treatment of achalasia due to its minimally invasive nature and high effectiveness in improving the patient’s quality of life.^[32,33]^ The number of cases in which endoscopists is proficient in POEM surgery is 12 to 14.^[34]^ In addition to achalasia, POEM is also a favorable safe and efficient procedure for patients with advanced achalasia featured with megaesophagus and/or sigmoid-shaped esophagus.^[35]^ Moreover, POEM can be a new option for the treatment of esophageal motility disorders, including esophagogastric junction outflow obstruction, distal esophageal spasm, etc.^[36]^ The high incidence of gastroesophageal reflux disease after POEM has been a topic. POEM with fundoplication is a novel natural orifice transluminal endoscopic surgery procedure to prevent the high incidence of reflux-related adverse events.^[37]^

There were some limitations in this study. First, we included only English literature from the Web of Science Core database in this study, which could lead to incomplete database retrieval and ignore a large number of studies published in other languages. Second, we included only articles and reviews from the Web of Science Core database in this study, which could lead to incomplete literature analysis. All of these could cause bias.

Overall, this study used a bibliometric approach to analyze the literature in the field of POEM. Research on POEM has significantly increased in the last decade globally and it will continue to increase. Meta-analysis, high-resolution esophageal manometry, geriatric patient, third space endoscopy, adverse event, and gastric peroral endoscopic myotomy might be the latest research frontiers and should receive more attention.

## Author contributions

**Conceptualization:** Xiaowei Tang.

**Data curation:** Qingliang Zhu, Han Zhang, Peiling Gan.

**Formal analysis:** Qingliang Zhu, Han Zhang, Shu Huang, Ruiyu Wang.

**Investigation:** Peiling Gan, Ruiyu Wang.

**Methodology:** Shu Huang, Peiling Gan.

**Resources:** Ruiyu Wang.

**Software:** Yan Peng.

**Supervision:** Yan Peng, Muhan Lü.

**Validation:** Yan Peng, Muhan Lü.

**Visualization:** Yan Peng.

**Writing – original draft:** Qingliang Zhu, Han Zhang.

**Writing – review & editing:** Xiaowei Tang.
